# Prevalence and comorbidity of osteoporosis– a cross-sectional analysis on 10,660 adults aged 50 years and older in Germany

**DOI:** 10.1186/s12891-018-2060-4

**Published:** 2018-05-14

**Authors:** Marie-Therese Puth, Manuela Klaschik, Matthias Schmid, Klaus Weckbecker, Eva Münster

**Affiliations:** 1 Institute of General Practice and Family Medicine, University of Bonn, Sigmund-Freud-Straße 25, 53127 Bonn, Germany; 2 Department of Medical Biometry, Informatics and Epidemiology, University Hospital Bonn, Sigmund-Freud-Straße 25, 53127 Bonn, Germany

**Keywords:** Prevalence, Socioeconomic level, Comorbidity, Osteoporosis, Multimorbidity, Germany

## Abstract

**Background:**

Knowledge on prevalence of osteoporosis stratifying for socioeconomic background is insufficient in Germany. Little is known in Europe about other diseases that go along with it although these aspects are important for implementing effective public health strategies.

**Methods:**

This cross-sectional analysis was based on the national telephone survey “German Health Update” (GEDA 2012) performed in 2012/2013. GEDA 2012 provides information on self-reported diseases and sociodemographic characteristics for nearly 20,000 adults. Descriptive statistical analysis and multiple logistic regression were used to examine the association between osteoporosis and age, sex, other diseases and education defined by ISCED. Analyses were limited to participants aged 50 years and older.

**Results:**

Overall, 8.7% of the 10,660 participants aged 50+ years had osteoporosis (men 4.7%, women 12.2%). More than 95% of the adults with osteoporosis had at least one coexisting disease. The odds for arthrosis (OR 3.3, 95% CI 2.6-4.1), arthritis (OR 3.0, 95% CI 2.2-4.2), chronic low back pain (OR 2.8, 95% CI 2.3-3.5), depression (OR 2.3, 95% CI 1.7-3.1) and chronic heart failure (OR 2.3, 95% CI 1.6-3.1), respectively, were greater for adults with osteoporosis. Education showed no significant association with osteoporosis.

**Conclusions:**

There was no clear evidence of socioeconomic differences regarding osteoporosis for adults in Germany. However, clinicians need to be aware that multimorbidity is very common in adults with osteoporosis. Health care interventions for osteoporosis could be improved by offering preventive care for other diseases that go along with it. Over- or under-diagnosis in different socioeconomic levels has to be further explored.

## Background

Osteoporosis and its consequences are a major public health concern and amount in high expenses for health care systems [1, 2]. For the affected patients it results in serious impairment in quality of life [2]. The World Health Organization (WHO) estimates the lifetime risk in a developed country for an osteoporotic fracture of hip, vertebra or wrist at 30-40% [3]. Exact data of the prevalence and comorbidities for the German population are rare. The “European Prospective Osteoporosis Study” (EPOS) stated a prevalence of 15% in women aged 50-60 years und 45% in women older than 70 years. In men the prevalence was 2.4% at age 50-60 years and 17% in men older than 70 years [4]. In total numbers this sums up to an estimated 4-7 million people with osteoporosis in Germany [4]. As the population structure is constantly changing towards a higher median age the overall share of osteoporosis patients is expected to grow continuously.

We wanted to take a closer look at the prevalence of osteoporosis in Germany, stratifying not only for more narrowly defined age groups and sex but also for socioeconomic level. This analysis is of high interest as the link between socioeconomic level and health and health behaviour is well documented [5–8] but is still lacking for osteoporosis [9, 10].

Often, bone mineral density (BMD) measurements alone are used to diagnose osteoporosis and/or to assess the chance of fractures. Beyond BMD, however, there are additional factors that similarly contribute to the disease. In addition to unchangeable factors as female gender, age, ethnicity or family history of fractures many preventable factors as poor lifestyle habits or physical inactivity have a significant impact on osteoporosis and fracture risk [11–13].

When discussing the health problems of osteoporosis patients the main focus is usually directed towards bone fractures as these are the most immediate consequences of the disease. Little is known about other diseases that go along with osteoporosis and equally impair the patients’ quality of life. Examining the association of osteoporosis with a range of different medical conditions might help to improve the health care for affected patients by offering early or even preventive care for diseases that go along with it.

## Methods

Our analysis was based on the Public Use File (PUF) of the national telephone health interview survey “German Health Update” (GEDA 2012) conducted by the Robert Koch Institute between March 2012 and March 2013 [14]. The Robert Koch Institute is a federal institution financed by the German Federal Ministry of Health that in addition to the research of infectious diseases is responsible for analysing national long-term public health trends [15]. As part of the health monitoring, the cross-sectional survey GEDA 2012 collected information about a range of health related topics involving current health conditions and medical history as well as sociodemographic characteristics [16]. The target population included fluently German-speaking adults of at least 18 years of age who were living in private households with landline telephone. Using a two-stage sampling procedure, the ADM-Sampling-System covered all possible phone numbers in Germany and was applied for the selection at household level [17, 18]. Random sampling at the individual level was performed by the Kish selection grid method that randomly selected an adult aged 18+ years out of all adults aged 18+ years in a private household [19, 20]. 19,294 participants completed the computer assisted telephone interviews (CATI) which matches a ‘cooperation rate at respondent level’ of 76.7% and a ‘response rate 3’ of 22.1% using standards of the American Association for Public Opinion Research [16, 20, 21]. The study involved the use of a previously-published de-identified database (secondary data analysis) so ethics approval and participant consent was not necessary [22].

The PUF contains data on survey participants in an anonymous form and provides information on self-reported health conditions including osteoporosis and 14 other medical diagnoses. The analyses were limited to participants aged 50 years and older as only those were asked about a medical history of osteoporosis [20]. Specifically, participants were asked “Have you ever been diagnosed with osteoporosis, also referred to as bone loss, by a physician?”. If it was affirmed, they were asked “Have you been diagnosed with osteoporosis in the last 12 months?”

To assess current health conditions, we considered only participants that stated suffering from osteoporosis in the past 12 months. The same criterion was used for any of the other medical diagnoses, namely hypertension, chronic heart failure, diabetes mellitus, bronchial asthma, hypercholesterolemia, chronic bronchitis, chronic liver disease, arthrosis, arthritis and depression. Lifetime history was only assessed for four diagnoses associated with long-term damages (coronary heart disease, myocardial infarction, cancer and stroke). In addition, data on self-reported chronic low back pain for at least 3 months was considered. Details on the exact definitions of the aforementioned diseases have already been published [20].

For age-specific analyses, 5-year age groups were used that were given by 50-54 years, 55-59 years, 60-64 years, 65-69 years, 70-74 years, 75-79 years, 80-84 years and 85 years or older. Information on educational qualification according to the International Standard Classification of Education (ISCED 1997) was summarized into low education (level 1, 2), medium education (level 3A, 3B, 4A) and high education (level 5A, 5B, 6) [20]. The Body Mass Index (BMI) estimated by self-reported body height and weight of each respondent was classified to underweight (BMI < 18.5 kg/m^2^), normal (18.5 kg/m^2^ ≤ BMI < 25 kg/m^2^), overweight (25 kg/m^2^ ≤ BMI < 30 kg/m^2^) and obese (BMI ≥ 30 kg/m^2^) according to WHO’s criteria [23]. Alcohol consumption was assessed using the “Alcohol Use Disorders Identification Test-Consumption” (AUDIT-C) [20, 24] and was categorized in no alcohol consumption, moderate alcohol consumption and high alcohol consumption [20]. Self-reported smoking status was summarized into non-smoker, ex-smoker or current smoker (daily or occasional) [20].

Prevalence rates of osteoporosis with 95% confidence intervals (CI) were determined for the total cohort aged at least 50 years as well as for subgroups defined by sex, age, education, BMI, smoking status and alcohol consumption. To correct for any deviations of the GEDA 2012 study population from the German population, prevalence rates were weighted according to the standardized weighting factor based on age, sex, education and residential region provided by the Robert-Koch Institute [20]. The unweighted number of participants in each subgroup is also displayed. Based on multiple logistic regression, odds ratios (OR) with 95% confidence intervals were computed to evaluate associations between other medical diagnoses and osteoporosis adjusted for age, sex, education, BMI, smoking status and alcohol consumption. For all independent variables in multiple regression analysis, the amount of missing responses did not exceed 2% hence missing responses were allocated to the reference category. Additional sensitivity analyses restricted to participants with valid data on all independent variables (complete cases) were performed. All analyses were realized using IBM SPSS Statistics (version 24) [25] with the complex sample module and R (version 3.3.3) [26].

## Results

The total number of participants aged 50 years and older was 10,744. Of those, 84 participants were excluded from the analysis due to unknown or missing responses regarding a diagnosis of osteoporosis. Thus, the present study included data of 10,660 participants (in the following termed “study population”) of which 911 stated suffering from osteoporosis within the past 12 months (hereinafter referred to as “osteoporosis population”). Sociodemographic characteristics of the study and osteoporosis population are summarized in Table 1.

**Table 1:** Sociodemographic characteristics by osteoporosis including adjusted odds ratios and 95% confidence intervals (GEDA 2012)

Characteristics	Study population n (%^a^)	%^a^ with Osteoporosis (95% CI)	Osteoporosis population n (%^a^)	OR^b^ (95% CI)
Total	10,660 (100)	8.7 (8.0-9.6)	911 (100)	
Sex***				
Male	4961 (46.5)	4.7 (3.8-5.9)	193 (25.2)	ref.
Female	5699 (53.5)	12.2 (11.1-13.5)	718 (74.8)	2.3 (1.7-3.0)
Age groups*** (years)				
50-54	2147 (19.1)	3.2 (2.4-4.2)	62 (6.9)	ref.
55-59	1429 (16.4)	6.3 (4.7-8.5)	87 (11.9)	2.1 (1.4-3.3)
60-64	1928 (14.6)	5.8 (4.5-7.5)	119 (9.7)	2.0 (1.3-3.0)
65-69	1378 (12.0)	8.7 (6.6-11.3)	124 (11.9)	2.9 (1.9-4.5)
70-74	1645 (15.0)	11.3 (8.9-14.2)	159 (19.4)	4.2 (2.8-6.3)
75-79	1252 (13.4)	12.5 (10.2-15.3)	187 (19.2)	4.2 (2.8-6.3)
80-84	588 (6.6)	17.8 (14.1-22.3)	113 (13.5)	5.6 (3.6-8.7)
85+	293 (2.8)	23.3 (16.8-31.5)	60 (7.5)	7.5 (4.4-12.8)
Level of education				
High	4816 (22.7)	5.5 (4.9-6.3)	318 (14.4)	ref.
Medium	5155 (54.1)	8.3 (7.5-9.2)	495 (51.4)	1.2 (1.0-1.5)
Low	674 (23.2)	13.0 (10.4-16.0)	98 (34.3)	1.3 (0.9-1.9)
BMI* (kg/m^2^)				
18.5 ≤ BMI < 25 (normal)	4015 (35.9)	10.8 (9.4-12.4)	396 (44.2)	ref.
18.5 > BMI (underweight)	112 (0.9)	16.9 (7.8-32.8)	19 (1.8)	1.4 (0.5-3.4)
25 ≤ BMI < 30 (overweight)	4290 (42.3)	7.4 (6.3-8.7)	318 (35.7)	0.8 (0.6-1.0)
30 ≥ BMI (obese)	2022 (20.9)	7.7 (6.2-9.5)	166 (18.3)	0.7 (0.5-0.9)
Smoking status				
Non-smoker	5220 (48.2)	10.3 (9.2-11.6)	524 (57.0)	ref.
Ex-smoker	3423 (31.4)	6.8 (5.7-8.2)	249 (24.6)	1.0 (0.8-1.3)
Current smoker	2014 (20.3)	7.9 (6.2-10.1)	138 (18.5)	1.3 (1.0-1.8)
Alcohol consumption*				
Moderate	5805 (52.3)	7.6 (6.7-8.7)	468 (46.4)	ref.
Never	2059 (23.5)	13.0 (11.1-15.2)	269 (35.3)	1.3 (1.0-1.6)
High	2716 (24.2)	6.5 (5.1-8.3)	164 (18.3)	0.9 (0.6-1.2)

^a^Weighted results to represent the adult population in Germany. Unweighted *n* may not add up to total *n* due to missing responses

^b^Odds ratios estimated from logistic regression adjusted for age, sex, education, BMI, smoking status and alcohol consumption, *** *p* < 0.001 ** *p* < 0.01 * *p* < 0.05. Missing responses were allocated to the reference category

**Fig. 1:**
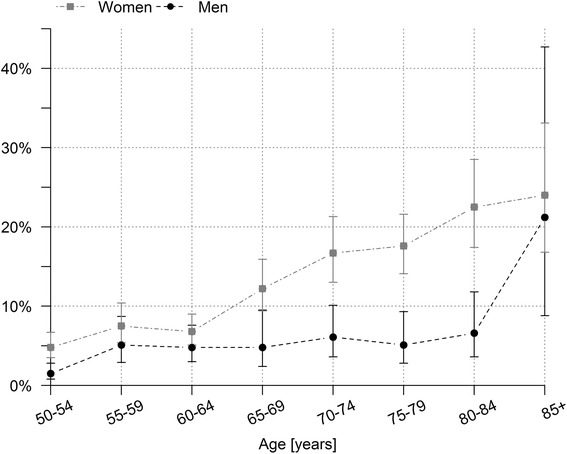
Age and sex-specific prevalence of osteoporosis with 95% confidence intervals (GEDA 2012)

In total, 8.7% (95% CI 8.0-9.6%) of the adult population aged 50 years and older had osteoporosis with significant differences between men (4.7, 95% CI 3.8-5.9%) and women (12.2, 95% CI 11.1-13.5%). The proportion of female adults with osteoporosis increased considerably with age; the prevalence of osteoporosis for men remained nearly unchanged until the age of 84 years (Fig. 1). Regarding the level of education, people with a low educational level showed higher prevalence rates of osteoporosis compared to those with a higher educational level. Overweight or obese adults had smaller prevalence rates than people with a BMI within the normal range. The prevalence of osteoporosis was higher for non-smokers in comparison to ex- and current smokers and participants with a moderate or high consumption of alcohol showed lower rates than respondents that stated to never drink alcohol (Table 1).

Age, sex, alcohol consumption and BMI showed a significant association with the odds for osteoporosis. The odds of having osteoporosis were higher for female adults than male adults. Using 50 - 54 years old adults as reference, the odds for osteoporosis increased with age. Overweight or obese adults were associated with lower odds for osteoporosis in comparison to adults with normal weight (Table 1).

More than 95% of the adults with osteoporosis had at least one comorbidity and about two thirds (65.7%) had three or more comorbid diseases. For adults without osteoporosis, only 80.6% reported at least one chronic condition and 39.2% had three or more different chronic diseases (data not shown). As illustrated in Table 2, arthrosis (63.2%) was the most common comorbidity among participants with osteoporosis followed by hypertension (51.3%), chronic low back pain (49.6%) and hypercholesterolemia (38.6%). About one in every five adults with osteoporosis suffered from coronary heart disease (21.0%) or arthritis (20.6%). In line with this, hypertension (44.2%), arthrosis (34.5%), hypercholesterolemia (30.1%) and chronic low back pain (26.8%) were also the most frequent conditions in the study population but they were followed by diabetes mellitus (13.7%), coronary heart disease (13.7%) and any type of cancer (12.2%).

**Table 2:** Associations between osteoporosis and comorbidities with adjusted odds ratios and 95% confidence intervals (GEDA 2012)

Comorbidity	Study population n (%^a^) (*N* = 10,660)	Osteoporosis population n (%^a^) (*N* = 911)	OR^b^ (95% CI)
Arthrosis	3541 (34.5)	573 (63.2)	3.3 (2.6-4.1)***
Hypertension	4602 (44.2)	464 (51.3)	1.2 (1.0-1.5)
Chronic low back pain	2656 (26.8)	442 (49.6)	2.8 (2.3-3.5)***
Hypercholesterolemia	3092 (30.1)	334 (38.6)	1.5 (1.2-1.8)**
Coronary heart disease	1278 (13.7)	175 (21.0)	1.5 (1.1-2.0)**
Arthritis	799 (8.3)	171 (20.6)	3.0 (2.2-4.2)***
Any type of cancer	1334 (12.2)	154 (16.0)	1.2 (0.9-1.6)
Depression	911 (8.8)	128 (15.3)	2.3 (1.7-3.1)***
Chronic heart failure	600 (6.1)	110 (14.0)	2.3 (1.6-3.1)***
Diabetes mellitus	1418 (13.7)	122 (13.8)	0.9 (0.7-1.2)
Bronchial asthma	695 (7.1)	106 (12.2)	1.6 (1.2-2.2)**
Chronic bronchitis	701 (7.2)	119 (11.9)	1.6 (1.2-2.2)**
Stroke	437 (4.6)	67 (9.1)	1.8 (1.1-2.8)*
Myocardial infarction	596 (6.2)	59 (7.3)	1.0 (0.7-1.5)
Chronic liver disease	232 (2.1)	36 (3.8)	1.8 (1.0-3.2)*

^a^Weighted results to represent the adult population in Germany

^b^Odds ratio estimated from logistic regression adjusted for age, sex, education, BMI, smoking status and alcohol consumption, *** *p* < 0.001 ** *p* < 0.01 * *p* < 0.05. A separate regression model was fitted for each comorbidity

Eleven out of fifteen comorbidities showed a significant association with osteoporosis. Of note, for adults with osteoporosis, the odds for arthrosis, chronic low back pain, arthritis, depression and chronic heart failure, respectively, were more than two times greater than for adults without osteoporosis (Table 2).

Sex-stratified analyses as well as analyses restricted to participants with valid data on all independent variables in regression (complete cases) showed similar results to the main analysis (data not presented).

## Discussion

The underlying study provides representative data on prevalence rates and comorbidities of osteoporosis based on the German population aged 50 years and older. The overall prevalence was estimated to 8.7% (men 4.7%, women 12.2%) and, for women, the rates increased substantially with age. According to multiple regression analysis, osteoporosis was significantly related to age, sex, BMI and alcohol consumption while smoking status and education showed no significant association. Adults with osteoporosis showed more than twofold increased odds for arthrosis, arthritis, chronic low back pain, chronic heart failure and depression, respectively.

Results on prevalence rates are difficult to compare as international prevalence estimates of osteoporosis are mostly based on the assessment of bone mineral density measurements using the WHO’s criteria with T-scores [3, 27, 28]. However, our results agree well with those of other studies on osteoporosis [27–32]. Using data from the National Health and Nutrition Examination Survey 2005-2010 with BMD measurements [27], Wright et al. estimated an overall prevalence of osteoporosis of 10.3% (men 4.3%, women 15.4%) in adults aged 50 years and older in the United States that is similar to the overall prevalence of 8.7% (men 4.7%, women 12.2%) in the present analysis. In comparison to other German studies [29–32], results on prevalence rates vary with regard to the methodology of measuring osteoporosis as well. On the one hand, our results are in line with those obtained in the first wave of the “German Health Interview and Examination Survey for Adults” (DEGS1) [29]. Similar to GEDA 2012, DEGS1 provides nationally representative data on the health status of the adult population between 18 and 79 years of age and estimated a lifetime prevalence of osteoporosis (self-reported) for people aged between 50 and 79 years to 8.5% (3.2% men, 13.1% women) [29]. Little differences with regard to socioeconomic status and an association with age for women were reported, too [29]. On the other hand, considering a study based on routine data of a statutory health insurance, prevalence rates were found to be higher. The BEST study that in addition to a medical diagnosis also included information on osteoporosis-related prescriptions and fractures reported an overall prevalence of 14% (6% men, 24% women) for insured people aged at least 50 years in the year 2009 [30]. Deviating methodical procedures might be responsible for differences in prevalence. Results of studies examining the relationship between smoking and osteoporosis as well as alcohol consumption and osteoporosis including low BMD and fracture risk are inconsistent [33–37]. There was also no clear evidence of a relationship between osteoporosis and smoking in the present study. The association between educational level and osteoporosis/BMD remains inconclusive as well [9, 10, 38]. While the prevalence of osteoporosis was significantly lower for higher educated adults in comparison to adults with a low educational level, results of the present regression analysis revealed no significant effects.

Prevalence rates may be biased as a consequence of misclassification as our results are based on self-reported diagnoses that were not clinically verified. Since osteoporosis is not associated with any symptoms prior to a fracture and information on possible fractures were not available within GEDA, prevalence rates may be underestimated by not taking account of yet undiagnosed adults. On the other hand, considering arthritis, for example, prevalence rates may be overestimated as it is known that patients with other joint disorders often falsely state to suffer from rheumatoid arthritis [20, 39].

Using self-reported information on sociodemographic characteristics such as BMI values may lead to biased estimates as well (reporting bias). Moreover, only adults living in private households were contacted, hospitalized adults or adults living in care homes could not be considered. As all interviews were carried out in German, adults had to speak and understand German, thus marginalized groups such as migrants could not be regarded [20]. Low-level educated adults agreed less often to participate in the telephone interview than people with a medium or high level of education [20]. A weighting factor provided by the Robert Koch Institute was used to approach the adult residential population structure in Germany [20].

Osteoporosis represents a major public health concern and its prevention is crucial to the maintenance of health [40]. It is a systemic condition characterized by changes in bone microarchitecture and a reduction of bone mass, both of which lead to decreased bone strength and at the same time to increased fracture risks. As a consequence, treatment at all ages aims at retaining bone mass to prevent any type of fracture (e.g. hip, spine). Fractures with severe complications are serious consequences of osteoporosis that have an influence on morbidity, functional impairment of health, a decrease in quality of life as well as an increase in medical costs [40, 41]. Additionally, at the time of a fracture, comorbidities in osteoporosis patients play a key role. Further, drug-drug interactions may affect the progress of the disease. Regarding osteoporosis, especially the consumption of drugs that have an effect on bone metabolism is of interest. In GEDA however, data on the use of pharmaceuticals were not collected and an evaluation of the use of different drug classes could therefore not be done.

In the present study nearly all adults with osteoporosis reported at least one comorbid condition, but the cross-sectional design did not allow for an analysis of cause and effect. In the GEDA study population participants that stated to suffer from osteoporosis were for example more than twice as likely to also suffer from depression. Drosselmeyer et al. showed that typically depression follows osteoporosis, but not vice versa [42]. Physical disability following fractures affects the capacity for independent living and complicates social participation. Besides, as physical activity is reduced in depressive patients but important to improve or at least stabilize bone mineral density, it would be important to recognize and treat the disease early. Of interest is also the association between arthrosis and osteoporosis. In the present study, participants with osteoporosis showed more than three times higher odds of having arthrosis. However, in most cross-sectional studies [43], arthrosis was negatively connected with osteoporosis in the sense that people with arthrosis showed higher BMD. Despite this negative association, the risk of osteoporotic fractures in patients with arthrosis remains the same [43]. Generally, arthrosis is associated with stiffness and pain in the affected joints, and this may reduce physical activity, which subsequently leads to instability and higher fracture risks. Hence, the relation of osteoporosis and arthrosis appears to be very complex and needs to be analysed further.

## Conclusions

The disease burden in adults with osteoporosis is of high relevance. Physicians need to be aware of the high occurrence of multimorbidity in adults with osteoporosis. Health care interventions for affected patients should be expanded by offering early or even preventive care for other diseases that go along with it. Over- or under-diagnosis in different socioeconomic levels has to be further explored.

## Abbreviations

ADM: Arbeitskreis Deutscher Markt- und Sozialforschungsinstitute e. V
AUDIT-C: Alcohol Use Disorders Identification Test-Consumption
BMD: Bone Mineral Density
BMI: Body Mass Index
CI: Confidence Intervals
DEGS: German Health Interview and Examination Survey for Adults
EPOS: European Prospective Osteoporosis Study
GEDA: German Health Update
ISCED: International Standard Classification of Education
OR: Odds Ratio
PUF: Public Use File
WHO: World Health Organization
